# Understanding the association of calligraphy activities with flow experience, peace of mind, and self-focused attention among university students

**DOI:** 10.3389/fpsyg.2025.1525503

**Published:** 2025-05-22

**Authors:** Jianing Wang, Kaizhi Tang

**Affiliations:** ^1^College of Art and Design, Hunan First Normal University, Changsha, China; ^2^Shanghai Academy of Fine Art, Shanghai University, Shanghai, China

**Keywords:** calligraphy activities, flow experience, peace of mind, self-focused attention, university students

## Abstract

**Introduction:**

Calligraphy, as a mindful practice, fosters focus, creativity, and relaxation, all of which contribute to a more peaceful mental state. For university students, regular engagement in calligraphy can help develop better coping mechanisms for academic and personal stress. This enhanced ability to manage stress can lead to improved mental well-being, greater self-awareness, and a more positive outlook, supporting their overall academic and personal development.

**Methods:**

This study employed convenience sampling and snowball sampling to select 453 university students from Hunan Province, China, in September 2024 as valid samples. AMOS v.23 was used to construct a structural equation model to validate the hypotheses.

**Results:**

The study revealed a significant positive relationship between calligraphy activities and both flow experience and peace of mind. Furthermore, a strong positive correlation was observed between flow experience, peace of mind, and self-focused attention. In addition, flow experience and peace of mind were found to mediate the relationship between calligraphy activities and self-focused attention.

**Discussion:**

The findings highlight the positive role of calligraphy activities in enhancing university students’ mental well-being, particularly through promoting flow experience and peace of mind. These factors not only directly benefit self-focused attention but also serve as mediators, suggesting that calligraphy can be an effective intervention for improving concentration and emotional balance. This study underscores the potential of incorporating calligraphy into student wellness programs to support both academic performance and personal development.

## 1 Introduction

With the accelerating pace of society and increasing academic competition, university students are facing increasingly severe mental health challenges (Liu et al., 2024). Numerous surveys conducted both domestically and internationally show that psychological pressure and emotional distress have become common and significant issues faced by university students (Yuan et al., 2022). According to the *2022 Survey Report on the Mental Health of Chinese College Students*, jointly published by the Institute of Psychology of the Chinese Academy of Sciences and Social Sciences Academic Press, 16.54% of undergraduates are at risk of mild depression, with 4.94% suffering from severe depression. 38.26% of students experience mild anxiety, and 7.02% are facing moderate to severe anxiety, with the prevalence of psychological issues increasing year by year (Yuan et al., 2022; Yang and Yang, 2022). Meanwhile, a 2023 survey by the American College Health Association (ACHA), involving over 55,000 undergraduates, showed that about 76% of students are experiencing moderate to severe psychological distress. Nearly half of the students reported experiencing high stress in the past month, over one-third had been diagnosed with anxiety, 28% suffered from depression, and about one-third had suicidal thoughts. These data collectively indicate that mental health issues among university students have become a critical issue in global higher education systems, necessitating the search for scientific, effective, and easily accepted intervention methods.

Although most universities have psychological counseling centers and offer various psychological support services, traditional intervention methods still face limitations in practice due to some students’ resistance to seeking help, as well as a lack of sufficient psychological literacy and the ability to express themselves (Liu and Li, 2023; Zheng and Ji, 2021). Surveys have found that many students are somewhat resistant to psychological counseling, particularly when it comes to intensive interventions in specific cases (Wei and Long, 2021). Furthermore, traditional psychological interventions often require long-term follow-up by professionals, and for some students, the participation threshold is relatively high. Therefore, exploring intervention methods that are culturally resonant, approachable, and have a low participation threshold has become an important direction for promoting mental health.

In this context, calligraphy, as a comprehensive art form that integrates visual elements, physical movements, and emotional regulation, has attracted increasing attention from researchers (Chu et al., 2018; Xu and Shen, 2023). Calligraphy is not only an important component of Chinese traditional culture, with profound cultural connotations and aesthetic value, but also its cultural function of fostering mental calmness and nurturing temperament makes it a potentially powerful tool for psychological health interventions (Hsiao et al., 2023). Recent studies have shown that calligraphy activities have positive effects in reducing anxiety, improving attention, and promoting emotional regulation. For example, Chu et al. (2018) found through empirical research that practicing calligraphy significantly reduces participants’ anxiety levels and improves their emotional states. Furthermore, the process of practicing calligraphy emphasizes concentration, relaxation, and creativity, which can effectively help individuals relieve psychological stress in daily life and facilitate emotional regulation (Zhang et al., 2021). Compared to certain high-cost or professionally-dependent psychological interventions, calligraphy offers greater accessibility and flexibility, making it easier to implement in campus environments with a low threshold, thereby expanding the coverage of psychological interventions (Yue et al., 2023).

To further explore the psychological effects of calligraphy activities, this study introduces the psychological concept of “flow experience” to investigate its role in the practice of calligraphy. Flow experience refers to the psychological state in which individuals are fully engaged in an activity, their consciousness highly focused, their sense of time diminished, and they experience great satisfaction and pleasure (Tse et al., 2020). Research indicates that when individuals are in a flow state, their anxiety levels decrease significantly, while their concentration and sense of well-being increase (Czikszentmihalyi, 1990). Flow experience helps enhance an individual’s sense of self-efficacy, making it particularly suitable for improving the psychological resilience and emotional regulation abilities of adolescents and university students (Kwok et al., 2011). The practice of calligraphy, with its unique rhythm, creativity, and immersive qualities, provides ideal conditions for individuals to enter a flow state. For instance, in calligraphy practice, students not only experience deep immersion in focusing on each stroke but also find emotional release and tranquility through this rhythmic activity, which effectively alleviates anxiety and enhances their sense of self-control and satisfaction (Yue et al., 2023; Wang and Tang, 2024).

In recent years, although numerous studies have explored various psychological interventions for university students, there remains a significant gap in the understanding of the role that artistic practices, such as calligraphy, play in promoting psychological well-being. While traditional psychological interventions, including counseling services, are widely available on university campuses, many students are reluctant to seek help due to social stigma, limited psychological literacy, or a lack of appropriate emotional expression skills (Wei and Long, 2021; Zheng and Ji, 2021). Furthermore, there is limited empirical research on alternative, low-barrier interventions that could offer both psychological benefits and cultural relevance. While existing studies have highlighted the benefits of mindfulness, meditation, and physical activities, the specific effects of calligraphy, particularly through the lens of flow experience, remain under-explored. Addressing this gap is crucial for broadening the scope of available interventions that can be seamlessly integrated into campus life and appeal to students’ diverse needs.

The purpose of this study is to empirically investigate the potential relationships and mechanisms between calligraphy, flow experience, and inner calmness as part of psychological health interventions for university students. Despite the growing interest in alternative interventions, limited research has specifically examined how engaging in artistic activities such as calligraphy can promote mental well-being. This study aims to fill this gap by exploring how the practice of calligraphy can facilitate a flow experience, which in turn may help students regulate emotions, reduce anxiety, and enhance overall psychological resilience. Through this empirical approach, the research seeks to provide a clearer understanding of how calligraphy can serve as an accessible, culturally relevant, and low-cost intervention for supporting student mental health.

The significance of this research lies in its potential to offer practical insights into a unique and effective method of promoting psychological well-being among university students. By incorporating calligraphy into mental health interventions, universities may have the opportunity to introduce a widely accessible, non-stigmatizing practice that resonates with students’ cultural values and personal interests. Ultimately, this study aims to contribute to the development of more diverse and inclusive strategies for mental health promotion, offering universities a valuable tool to foster a supportive and resilient student community.

The paper is organized into the following sections: Section 2 outlines the hypotheses and conceptual models. Section 3 details the methods used for data collection and analysis. Section 4 presents the results of the data analysis and tests the proposed hypotheses. Section 5 provides a discussion, including theoretical contributions, practical implications, and limitations, along with suggestions for future research. Finally, Section 6 offers the conclusion.

## 2 Literature review and hypothesis development

### 2.1 Flow theory

Flow theory was first introduced by Mihaly Csikszentmihalyi in 1975 to explore the intense focus and immense satisfaction experienced by individuals during certain activities (Beard, 2015). This flow state is often described as being in a “flow,” where individuals become fully immersed in the activity, feeling as if time has ceased to exist and external distractions are blocked out (Tse et al., 2020). This theory provides a robust framework for understanding psychological experiences in various activities, particularly in areas such as artistic creation, sports, and learning.

The core concepts of flow theory include the balance between challenge and skill, goal clarity, and intrinsic motivation (Abuhamdeh, 2020). Firstly, flow experiences occur when there is a dynamic balance between the challenge of the activity and the individual’s skill level (Tsaur et al., 2013). When the difficulty of the task is just right, individuals neither feel bored nor anxious; this balance is crucial for entering a flow state (Aust et al., 2022). Secondly, individuals need to have clear goals and receive immediate feedback while engaging in the activity. This sense of direction enhances focus on the task and increases engagement (Digutsch and Diestel, 2021). Lastly, flow experiences are intrinsically motivated, with satisfaction and a sense of accomplishment serving as the primary driving forces for participation (Lin et al., 2020).

Research indicates that flow experiences not only enhance individuals’ well-being and self-efficacy but also promote mental health. Many artistic activities, including calligraphy, painting, and music creation, can trigger flow experiences, helping participants alleviate anxiety and stress while enhancing emotional regulation abilities (Croom, 2015). Among university students, academic pressure and mental health issues are increasingly prominent. Related studies have found that participating in activities that induce flow experiences can help students improve their focus and coping abilities (Zheng and Ji, 2021). For instance, some research shows that students engaged in artistic creation activities exhibit significant improvements in both mental health and academic performance, closely linked to their ability to achieve a flow state during these activities (Zhang et al., 2024).

### 2.2 Hypothesis development

#### 2.2.1 Calligraphy activities, flow experience, and peace of mind

Calligraphy is a focused and creative art form that requires significant cognitive and emotional investment from its practitioners (Hsiao et al., 2023). Engaging in calligraphy is more than just an artistic endeavor; it demands attention, patience, and self-discipline, all of which create an ideal environment for the emergence of flow experiences. According to Csikszentmihalyi’s flow theory, a flow experience occurs when individuals are fully immersed in an activity, experiencing intense pleasure, satisfaction, and concentration (Tsaur et al., 2013). In the case of calligraphy, the clear goals—such as completing each stroke with precision and forming a harmonious piece of artwork—help participants achieve this deep state of immersion (Yue et al., 2023).

The flow experience is characterized by a heightened focus, where individuals lose track of time and become deeply absorbed in the task at hand (Aust et al., 2022). In calligraphy, this immersion is facilitated by the need for precision and the rhythmic nature of the strokes, which promote sustained concentration. As a result, participants often report feelings of intense satisfaction and fulfillment, which are essential elements of the flow experience. Furthermore, research has demonstrated that engaging in flow activities, such as artistic practices, can lead to improved psychological well-being by reducing stress and increasing self-efficacy (Mangialavori et al., 2024).

In addition, calligraphy is inherently meditative, encouraging participants to focus on the present moment and regulate their emotions. Peace of mind refers to a stable emotional state in which individuals experience calmness, clarity, and emotional balance (Wang and Tang, 2024). This psychological state is associated with reduced anxiety and improved cognitive function. When engaging in calligraphy, participants experience a reduction in external distractions, which allows them to enter a relaxed state (Kao et al., 2021). This mental clarity helps regulate emotions and promotes a sense of inner peace, which is why calligraphy can be considered a form of emotional self-regulation (Wang and Tang, 2024).

Thus, based on the theoretical foundations of flow and emotional regulation, we propose that calligraphy activities facilitate both flow experiences and peace of mind, which are central to improving participants’ mental well-being. Therefore, this study proposes the following hypotheses:

*Hypothesis 1 (H1)*: Calligraphy activities have a positive and significant association with flow experience.

*Hypothesis 2 (H2)*: Calligraphy activities have a positive and significant association with peace of mind.

#### 2.2.2 Flow experience and peace of mind

Flow experience and peace of mind are both essential dimensions of positive mental functioning, yet they reflect different psychological states. Flow experience, as conceptualized by Czikszentmihalyi (1990), refers to a state of optimal immersion and intrinsic engagement, in which individuals experience focused attention, effortless involvement, and a loss of self-awareness. During flow, individuals may perceive time as moving rapidly and derive deep satisfaction from the activity itself (Peifer et al., 2014). This state typically arises when there is a perceived balance between the challenges of a task and an individual’s skills (Digutsch and Diestel, 2021).

On the other hand, peace of mind refers to a stable emotional and psychological state characterized by calmness, clarity, and reduced anxiety (Lee et al., 2013). It involves a sense of mental stillness and emotional equilibrium, where individuals feel attuned to their inner needs and less reactive to external stressors (Wang and Tang, 2024). Unlike flow experience, which is primarily related to an active state of engagement, peace of mind is a more passive state of emotional and mental relaxation, often achieved through mindfulness or meditative activities (Liao et al., 2023; Sun et al., 2024). While flow experience is associated with heightened focus and performance, peace of mind is linked to a sense of inner calm and psychological comfort.

Although they represent different psychological states, flow experience and peace of mind are not mutually exclusive; instead, they often reinforce each other. Engaging in activities that elicit flow experience, such as calligraphy, can reduce stress and foster a quiet mental space conducive to peace of mind. The immersive and intrinsically rewarding nature of flow experience helps individuals temporarily disengage from daily worries, thus creating conditions that support a tranquil emotional state. In support of this perspective, existing studies have described the relationship between flow experience and peace of mind as synergistic (Mangialavori et al., 2024; Peifer et al., 2014; Tsaur et al., 2013). Flow experience, by fostering a deep state of concentration and engagement, enables individuals to temporarily detach from external pressures, contributing to mental clarity and emotional steadiness. Empirical evidence further indicates that individuals who frequently experience flow tend to report higher levels of psychological well-being, such as reduced anxiety and enhanced emotional balance (Aust et al., 2022). Therefore, especially in contexts like calligraphy—where focused attention and a calm mental state are essential, which flow experience may serve as a meaningful pathway to achieving peace of mind.

*Hypothesis 3 (H3)*: Flow experience has a positive and significant association with peace of mind.

#### 2.2.3 Flow experience, and peace of mind and self-focused attention

Flow experience is a state of deep psychological engagement in which individuals become fully immersed in an activity, experiencing high levels of concentration, intrinsic motivation, and a loss of awareness of time and external surroundings (Peifer et al., 2014). In the practice of calligraphy, flow can emerge as individuals concentrate on the fine details of each stroke and maintain a high level of perceptual and motor coordination. This intense engagement encourages inward attentional focus, prompting individuals to attend closely to their own bodily movements, thoughts, and experiences during the process. As such, flow experience is believed to enhance self-focused attention, which refers to the deliberate direction of cognitive resources toward one’s internal states (Hong et al., 2024; Aust et al., 2022). Flow also promotes attentional stability, enabling individuals to concentrate more effectively and sustain a heightened awareness of their internal experiences (Brosch et al., 2013; Córdova et al., 2023).

Peace of mind is characterized by a stable psychological state marked by clarity, calmness, and reduced reactivity to external stressors (Lee et al., 2013). In calligraphy practice, the repetitive and rhythmic nature of brushwork, as well as the aesthetic and contemplative qualities of the activity, may promote inner calm. This sense of tranquility reduces the interference of environmental distractions and facilitates deeper engagement with one’s internal thoughts and feelings. Studies have shown that individuals who experience peace of mind tend to exhibit stronger attention control and higher levels of self-awareness, which are essential components of self-focused attention (Zhang et al., 2021; Shields et al., 2020; Vago and Zeidan, 2016). Both flow experience and peace of mind are viewed as conducive to enhancing self-focused attention. While flow allows individuals to become absorbed in the process and heightens their focus on internal actions and sensations, peace of mind fosters a calm and distraction-free state in which individuals can further explore their inner thoughts and feelings. Calligraphy, by promoting both states, creates an environment that supports deeper engagement with the self.

Based on the theoretical and empirical findings, this study proposes the following hypotheses:

*Hypothesis 4 (H4)*: Flow experience has a positive and significant association with Self-Focused attention.

*Hypothesis 5 (H5)*: Peace of mind has a positive and significant association with Self-Focused attention.

#### 2.2.4 Mediation effects

Although no existing quantitative studies have explicitly examined the mediating effect of flow experience and peace of mind in the relationship between calligraphy activities and self-focused attention, there is a wealth of research exploring the individual relationships between these variables. Flow experience and peace of mind have both been studied extensively in various contexts, with evidence showing that they contribute to enhanced attention.

First, calligraphy is an activity that inherently demands high levels of concentration and skill, making it well-suited for inducing flow states (Yue et al., 2023). According to Czikszentmihalyi (1990), flow experience occurs when individuals are fully absorbed in an activity that presents optimal challenges aligned with their abilities. During calligraphy, individuals must attend to each stroke with precision, which fosters a state of deep concentration and sustained attention (Kao et al., 2021). Research has shown that engaging in such immersive tasks facilitates focused cognitive processing and reduces distractions from the external environment, thereby enhancing self-focused attention (Ming et al., 2021).

Second, the practice of calligraphy can cultivate a sense of peace of mind. The repetitive, deliberate, and aesthetically engaging nature of calligraphy encourages a calm and steady mental state. In this environment, individuals often report experiencing inner clarity and reduced mental noise, which allows for greater awareness of their internal experiences (Lee et al., 2013). Particularly among university students, who are frequently subject to academic and social pressures, calligraphy offers a quiet and contemplative activity that supports inward attention and reflective thinking (Wang et al., 2023). Peace of mind, therefore, provides a psychological foundation for enhancing self-focused attention through improved mental clarity and reduced interference from external distractions.

Therefore, this study proposes the following hypothesis. Flow experience and peace of mind are proposed to mediate the effect of calligraphy activities on self-focused attention. Specifically, calligraphy may influence self-focused attention in two ways. First, it may directly affect self-focused attention by requiring sustained cognitive engagement throughout the activity. Second, it may influence self-focused attention indirectly by fostering immersive psychological states, such as flow experience, and tranquil states, such as peace of mind, both of which support a deeper internal focus.

*Hypothesis 6 (H6)*: Flow experience and peace of mind mediate the relationship between calligraphy activities and Self-Focused attention.

**Figure 1:**
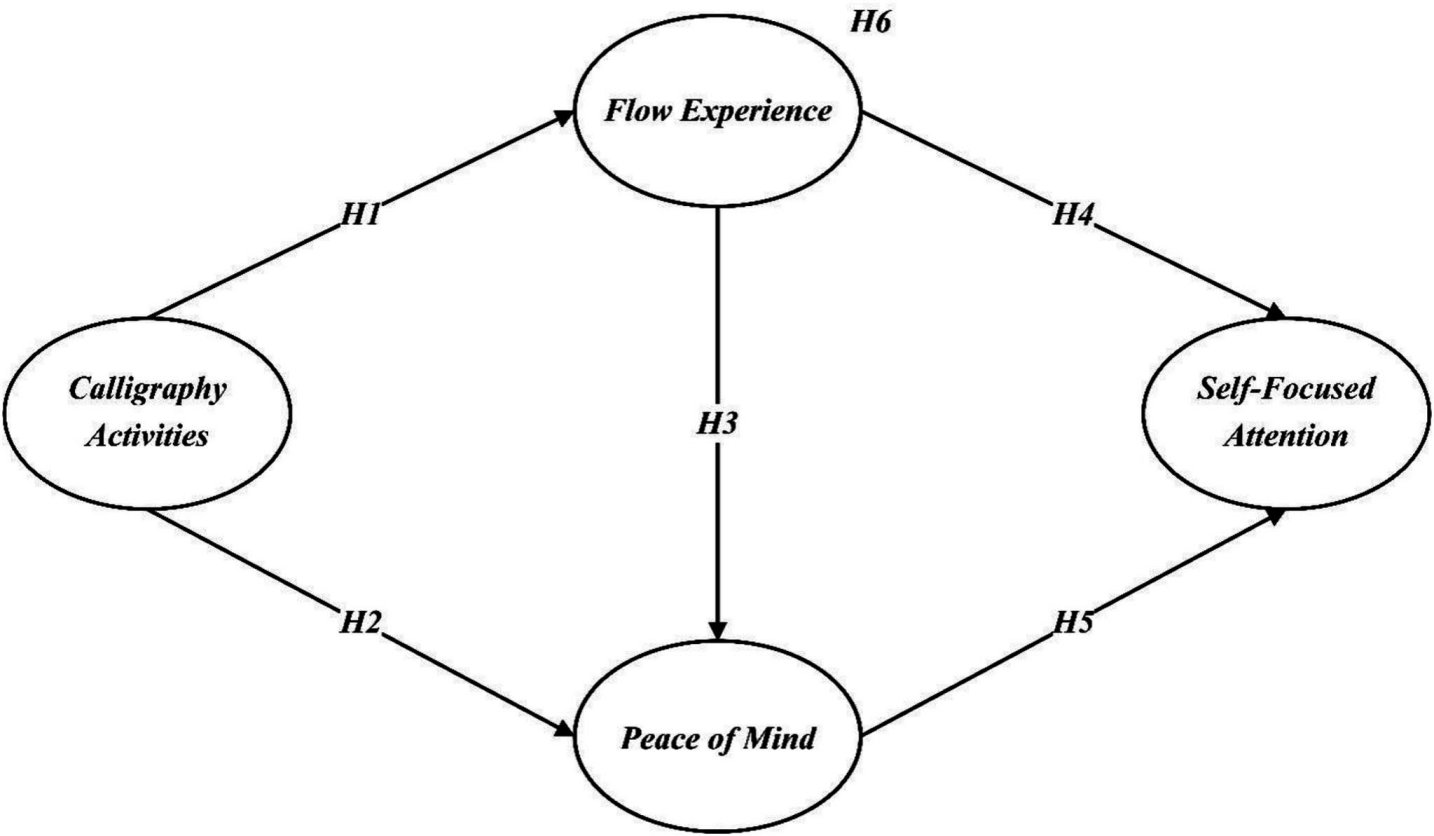
Hypothesis model.

All hypotheses are summarized in Figure 1.

## 3 Methodology

### 3.1 Participants and procedures

This study recruited university students from three universities in Hunan Province, China, as they are at a critical stage of psychological development, often facing academic pressure and challenges related to self-awareness. Calligraphy activities may have a positive impact on their mental health and concentration, making this population relevant for investigation.

Data collection was conducted via an online questionnaire survey in September 2024. To enhance sample diversity while minimizing bias, a combination of convenience sampling and snowball sampling was employed. Convenience sampling allowed for quick participant recruitment, while snowball sampling helped reach students from various academic and social backgrounds. All participants were informed of the study’s purpose, participated voluntarily, and were assured of anonymity and confidentiality to minimize response bias. After collecting 500 responses, invalid questionnaires were removed, resulting in 453 valid responses, yielding an effective response rate of 90.6%.

Table 1 presents the demographic characteristics of the sample. The gender distribution showed a higher proportion of female students (58.3%) compared to male students (41.7%). In terms of academic level, most participants were freshmen (39.7%) or seniors (34.0%). The major distribution indicated that 70.0% of students were from Arts & Humanities, while 30.0% were from Science & Engineering disciplines. Given the artistic nature of calligraphy, a higher representation of Arts & Humanities students was expected.

**Table 1:** Demographic characteristics (*n* = 453).

Profiles	Items	*n* (%)
Gender	Male	189 (41.7)
	Female	264 (58.3)
Academic year	Freshman	180 (39.7)
	Sophomore	73 (16.1)
	Junior	46 (10.2)
	Senior	154 (34.0)
Major	Arts & Humanities	317 (70.0)
	Science & Engineering	136 (30.0)
Do you have experience in calligraphy practice	Yes	389 (85.9)
	No	64 (14.1)

Additionally, 85.9% of participants reported prior experience with calligraphy, suggesting a high level of familiarity with the activity. While this enhances the study’s focus on psychological effects among experienced individuals, it may limit generalizability to those less familiar with calligraphy.

### 3.2 Instruments

The questionnaire consisted of five sections. The first section collected demographic information such as gender, grade, major, and whether the respondent had experience with calligraphy practice. The second section featured three items to assess participation in calligraphy activities over the past week (Wang and Tang, 2024), including the question, “How frequently did you practice writing last week?” These items were designed to capture participants’ recent engagement in calligraphy activities with clarity and simplicity. Responses were rated on a five-point Likert scale ranging from 1 (Never) to 5 (Always). The internal consistency of this section was good, with a Cronbach’s *α* of 0.934.

The third section measured flow experience using six items from Jackson et al. (2008), with sample questions like, “While performing, I have a good sense of how well I am doing.” The scale has been validated in previous studies and is suitable for assessing flow in the context of artistic practices. A five-point Likert scale was used (1 = Strongly Disagree, 5 = Strongly Agree), and the Cronbach’s α for this section was 0.929, indicating high internal consistency.

The fourth section, adapted from Lee et al. (2013), used five items to gauge peace of mind, such as, “I feel content and comfortable with myself in daily life.” This scale is widely used in studies on mindfulness and emotional well-being, with demonstrated reliability. Responses were rated on a five-point Likert scale (1 = Strongly Disagree, 5 = Strongly Agree), and Cronbach’s α for this section was 0.966.

The fifth section assessed self-focused attention with seven items from Bögels et al. (1996), including, “In the presence of others, I’m constantly focusing on whether my heart is beating.” The scale has been shown to effectively measure self-focused attention in various psychological contexts. It also employed a five-point Likert scale (1 = Strongly Disagree, 5 = Strongly Agree), and the Cronbach’s α was 0.955, indicating strong internal consistency.

### 3.3 Data analysis

This study utilized AMOS v.23 to build a structural equation model (SEM) examining how calligraphy activities help university students improve flow experience and peace of mind, ultimately influencing their self-focused attention. The SEM approach was chosen because it enables simultaneous analysis of multiple relationships among latent variables, providing a robust framework for testing the hypothesized construct model. Model parameters were estimated using the maximum likelihood (ML) method, following a two-step process to assess both the measurement and structural models. The measurement model evaluated the reliability and validity of the constructs, while the structural model tested the hypothesized relationships among the variables. Key aspects evaluated included the model’s reliability, validity, fit indices, path coefficients, and mediating effects.

To address potential common method variance (CMV) from self-reported behaviors, researchers followed the approach recommended by Mossholder et al. (1998). This approach involved applying Harman’s single-factor test to examine whether a single factor accounted for the majority of variance. Additionally, a marker variable was used to detect any significant CMV influence. A comparison of two models was conducted based on degrees of freedom and chi-square values. The first model had a chi-square value of 6257.811 with 209 degrees of freedom, while the second model had a chi-square value of 1013.488 with 183 degrees of freedom. Both models showed *p*-values below 0.001. These results confirm a good model fit, indicating that CMV is not a significant issue in this study.

## 4 Results

### 4.1 Measurement model

The reliability and validity of the latent variables were evaluated through confirmatory factor analysis (CFA) using AMOS v.23. All constructs showed strong internal consistency, with Cronbach’s *α* values exceeding 0.9 (Table 2), meeting the threshold recommended by Fornell and Larcker (1981). The average variance extracted (AVE) for each variable was above 0.6, and composite reliability (CR) was greater than 0.9, indicating good convergent validity. Additionally, factor loadings from principal component analysis ranged from 0.786 to 0.956 (Table 2), further supporting the construct validity of the measurement model. Discriminant validity was confirmed, as the square root of the AVE for each variable was higher than the correlations between constructs (Table 3).

**Table 2:** Reliability and validity.

Items	Factor loadings	Cronbach’s α	CR	AVE
Calligraphy activities (CA)		0.934	0.936	0.829
CA1	0.904			
CA2	0.954			
CA3	0.872			
Flow experience (FE)		0.929	0.930	0.688
FE1	0.838			
FE2	0.856			
FE3	0.802			
FE4	0.857			
FE5	0.785			
FE6	0.837			
Peace of mind (POM)		0.966	0.966	0.851
POM1	0.854			
POM2	0.941			
POM3	0.956			
POM4	0.914			
POM5	0.944			
Self-focused attention (SFA)		0.955	0.955	0.752
SFA1	0.832			
SFA2	0.909			
SFA3	0.892			
SFA4	0.896			
SFA5	0.853			
SFA6	0.859			
SFA7	0.833			

**Table 3:** Pearson correlation.

Construct	CA	FE	POM	SFA
CA	(0.910)			
FE	0.655 **	(0.829)		
POM	0.501 **	0.539 **	(0.922)	
SFA	0.232 **	0.342 **	0.422 **	(0.867)

The square root of the AVE is in diagonals; off diagonals are a Person’s corrections of contracts. ** *p* < 0.01.

### 4.2 Structural model

**Figure 2:**
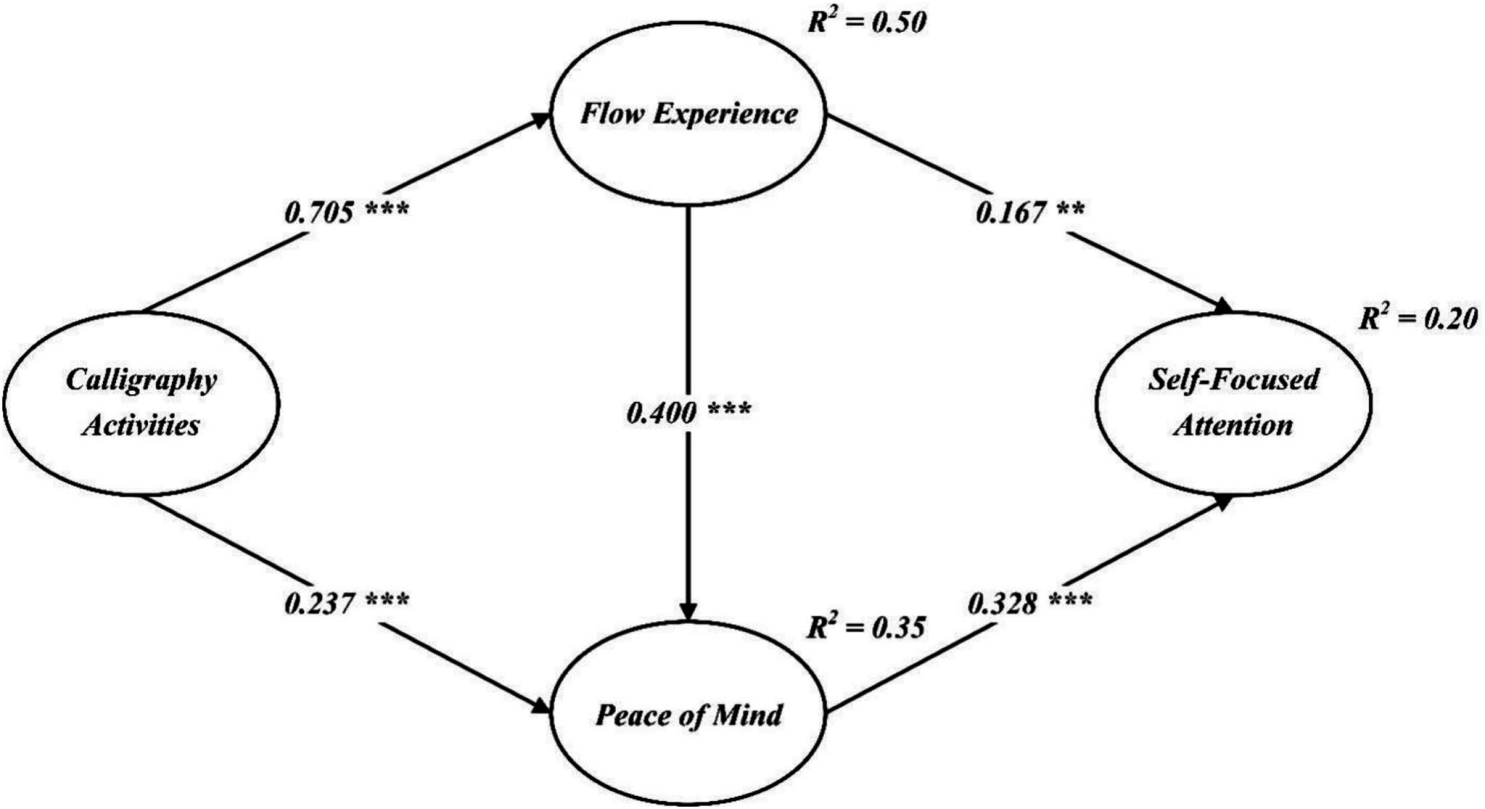
Structural path model. *** *p* < 0.001.

After confirming the reliability and validity of the measurement model, the structural model was tested using AMOS v.23 with 5,000 bootstrap samples. The CFA results indicated a good model fit (χ^2^/df = 3.665, NFI = 0.938, IFI = 0.954, TLI = 0.946, CFI = 0.954, RMSEA = 0.077), showing alignment with the data. Pearson correlations in Table 3 confirmed the relationships among the variables, and Figure 2 displays the standardized coefficients.

As shown in Figure 2, calligraphy activities were positively related to flow experience (*β* = 0.705, *p* < 0.001) and peace of mind (*β* = 0.237, *p* < 0.001), supporting H1 and H2. Flow experience also positively correlated with peace of mind (*β* = 0.400, *p* < 0.001) and self-focused attention (*β* = 0.167, *p* < 0.01), supporting H3 and H4. Peace of mind was also positively related to self-focused attention (*β* = 0.328, *p* < 0.001), supporting H5.

The mediating effects were examined using bootstrap estimation with 5,000 resamples and 95% bias-corrected confidence intervals, as presented in Table 4. The results indicated a significant indirect effect of calligraphy activities on self-focused attention, mediated by flow experience and peace of mind. This mediation was substantial, with an estimate of 0.288 [SE = 0.037, CI = (0.219, 0.365), *p* < 0.001], providing strong support for H6.

**Table 4:** Standardized indirect effect.

Indirect Effect	Point Estimate	Product of Coefficients		Bootstrapping		
				Bias-Corrected 95% CI		Two-Tailed Significance
		SE	*Z*	Lower	Upper	
CA → SFA	0.288	0.037	7.784	0.219	0.365	*p* < 0.001

## 5 Discussion

### 5.1 Theoretical contributions

This study makes several important theoretical contributions to the fields of educational psychology, art therapy, and mindfulness research. By conducting the first quantitative study on the relationship between calligraphy practice, flow experience, peace of mind, and self-focused attention, this research moves beyond confirming existing qualitative findings and provides a refined theoretical understanding of how traditional art forms interact with psychological constructs. Specifically, the study demonstrates how these constructs operate uniquely within the embodied and culturally embedded practice of calligraphy, offering new perspectives for the development of psychological theory.

First, while flow theory has been extensively applied in performance and learning contexts, this study extends the theory by situating it within the traditional, contemplative practice of Chinese calligraphy. It demonstrates that flow can emerge not only from externally goal-driven or high-stimulation activities but also from artistic practices that emphasize quiet, precise, and repetitive actions. This challenges the prevailing notion that flow predominantly arises from fast-paced or physically engaging tasks, and instead shows that immersive attention can be cultivated through aesthetic and culturally grounded routines. Thus, this research offers a theoretical expansion by illustrating how the nature and triggers of flow may vary in artistic versus performative domains.

Secondly, the study introduces a novel conceptual linkage between peace of mind and self-focused attention in the context of traditional arts. While peace of mind has been discussed within mindfulness and emotional regulation literature, its role as a distinct and measurable psychological state that mediates attention in creative contexts has not been fully articulated (Lin et al., 2020; Wang and Tang, 2024). By empirically validating its influence, this research repositions peace of mind not merely as a passive emotional state, but as an active cognitive-affective mechanism that facilitates internal focus. This reframing expands the theoretical utility of the construct, particularly in the domain of art-based interventions and contemplative education.

Moreover, the study innovatively combines the frameworks of flow theory and mindfulness theory to construct a dual-pathway mediation model, in which both flow experience and peace of mind mediate the effects of calligraphy activities on self-focused attention. This integrated approach offers a new explanatory mechanism that bridges the typically separate literatures on performance immersion (flow) and mental clarity (mindfulness). It demonstrates that these constructs do not operate in isolation, but may mutually reinforce each other when embedded in structured artistic activities. This synthesis thus proposes a new theoretical pathway, which is termed here as “aesthetic mindfulness,” wherein attentional control and emotional balance are achieved simultaneously through cultural creative practice.

Furthermore, this research challenges the traditional assumption within mindfulness theory that emotional regulation and self-awareness are primarily cultivated through static or meditative practices. It shows that dynamic, embodied art forms like calligraphy can generate similar psychological benefits, thereby expanding the scope of mindfulness-related constructs. In doing so, the study positions calligraphy not only as a cultural art form but as a psychologically rich activity that can serve as a vehicle for both cognitive and emotional development.

In conclusion, by exploring how flow experience and peace of mind mediate the relationship between calligraphy and self-focused attention, this study not only confirms prior assumptions but meaningfully extends theoretical understanding across multiple psychological frameworks. It provides new insight into how traditional artistic practices can be re-theorized as structured experiences that generate specific psychological outcomes. This contributes to an evolving discourse on the intersection of culture, cognition, and emotion, and offers valuable directions for future research in art-based therapy, contemplative education, and attention regulation.

### 5.2 Practical implications

The findings of this study highlight the significant role that calligraphy can play in enhancing university students’ self-focused attention, particularly through its effects on flow experience and peace of mind. Both universities and students can take practical steps to leverage calligraphy’s benefits for improving mental well-being and concentration.

To begin with, this research provides universities with actionable insights for expanding their mental health and wellness initiatives. Universities can incorporate calligraphy into their programs by offering calligraphy courses or workshops as part of stress management initiatives. While previous research has shown the positive effects of meditation and yoga on mental health (Norris et al., 2018; Shields et al., 2020), calligraphy represents a distinct and culturally rich alternative that combines creative engagement with attentional training. Unlike practices that emphasize stillness or passive observation, calligraphy fosters active focus through expressive movement, enabling students to enter a state of flow while enhancing their capacity for emotional regulation. By embedding calligraphy into either academic or extracurricular programming, universities can offer students a culturally meaningful and empirically supported method to cope with academic stress, manage emotions, and sharpen concentration.

Importantly, this study reframes calligraphy not merely as an art form but as a psychologically beneficial practice that promotes attentional and emotional self-regulation. While the mental health literature has largely centered on more mainstream interventions such as meditation, yoga, or cognitive-behavioral approaches, this research calls attention to the underexplored potential of traditional creative arts like calligraphy. The act of writing characters—when approached with focused engagement—enables students to cultivate peace of mind and sustained attention, thereby improving their ability to self-reflect and regulate inner states. Universities can therefore develop integrated programs that incorporate calligraphy training into wellness curricula, providing an alternative, culturally resonant strategy to support diverse student needs in a holistic manner.

For students, this study underscores the tangible value of engaging in calligraphy as part of their daily routine. Practicing calligraphy not only offers a break from academic pressures but also serves as a tool for enhancing focus, promoting relaxation, and stimulating creativity. Through the immersive experience of writing, students can achieve a deepened sense of presence and self-awareness, which are critical for sustaining attention and emotional balance. Participation in calligraphy groups, university-hosted workshops, or competitions can further reinforce these benefits. Such group settings not only encourage regular practice and social engagement but also foster a sense of shared purpose and motivation. Moreover, the social dimension of creative activities has been shown to support emotional resilience and provide peer-based support (Restrepo et al., 2023), amplifying the overall psychological benefits.

This study fills a notable gap in the existing literature by uncovering the mechanisms through which calligraphy enhances emotional regulation and self-focused attention. Although prior research has explored the benefits of meditation and art therapy (Vago and Zeidan, 2016), few studies have quantitatively examined how traditional cultural practices like calligraphy contribute to psychological well-being through flow experience and inner peace. By empirically verifying these relationships, this study adds new dimensions to flow theory and offers a culturally grounded, evidence-based intervention strategy for student mental health.

In conclusion, this study offers new approaches for university mental health programs, particularly by integrating calligraphy to achieve emotional regulation and improve self-focus. Universities can enhance their mental health offerings by including calligraphy, which provides effective tools for stress management and focus training. The combination of creative expression and attentional engagement in such practices can significantly improve student well-being and offers a theoretical foundation for future research and practical applications in this field.

### 5.3 Limitations

Firstly, this study employed convenience sampling and snowball sampling to recruit university students from Hunan Province, China. While these methods allowed for efficient data collection, they may introduce selection bias, as participants who voluntarily took part may have had a greater interest in calligraphy or mindfulness-related activities. This could limit the representativeness of the sample and the generalizability of the findings to broader student populations or individuals from different cultural and educational backgrounds. Future studies should consider random sampling or stratified sampling to improve the representativeness of the sample and reduce potential biases. Expanding the study to include students from different regions and universities would further enhance external validity. Future research should consider recruiting a more balanced sample with varying levels of calligraphy experience to examine potential differences in psychological outcomes.

Secondly, the study relied solely on self-reported measures for calligraphy participation, flow experience, peace of mind, and self-focused attention. While self-reports provide valuable insights, they are susceptible to common method bias and social desirability bias, as participants may have overestimated their engagement in calligraphy or their psychological states. To address this, future research should incorporate objective measurements such as psychological well-being indicators, cognitive performance tests, physiological markers (e.g., heart rate variability, skin conductance), or behavioral observations. These empirical data sources could strengthen the validity of the findings by providing a more comprehensive assessment of calligraphy’s effects on psychological well-being and concentration.

Thirdly, this study adopted a cross-sectional design, which limits the ability to infer causality or examine the long-term effects of calligraphy activities on psychological well-being. While significant associations were observed, the temporal stability and sustained benefits of calligraphy remain unclear. Future research should conduct longitudinal studies to track changes in flow experience, peace of mind, and self-focused attention over time. Such an approach would provide deeper insights into whether regular engagement in calligraphy leads to lasting improvements in mental well-being.

Finally, this study did not explore prior experience with calligraphy as a potential moderating factor in its psychological effects. Individuals with different levels of familiarity or proficiency in calligraphy may experience varying degrees of psychological benefits. Future research could examine whether prior experience influences the strength of associations between calligraphy, flow experience, peace of mind, and self-focused attention. Understanding this moderating role could help refine intervention strategies and tailor calligraphy-based practices to different student populations.

## 6 Conclusion

This study investigated the impact of calligraphy activities on university students’ psychological well-being, specifically focusing on flow experience, peace of mind, and self-focused attention. The findings provide compelling evidence for the positive associations among these variables, highlighting the therapeutic potential of engaging in traditional art forms like calligraphy. The research established that participation in calligraphy significantly enhances flow experience, fostering creativity and a sense of fulfillment. As students immerse themselves in the intricate movements of calligraphy, they experience a shift in focus that allows them to transcend academic pressures, facilitating a deeper connection with themselves and their environment. Moreover, the study demonstrated that engaging in calligraphy contributes to a greater sense of peace of mind, helping to reduce stress and anxiety while improving emotional regulation. This finding is particularly relevant given the rising mental health challenges among university students, suggesting that creative practices may serve as effective coping strategies. Additionally, the results indicate that flow experience and peace of mind significantly influence self-focused attention, underscoring the importance of cultivating self-awareness. The implications of this research extend beyond theoretical contributions, offering practical insights for educational institutions to support student mental health through artistic engagement. By integrating such practices into psychological support programs, universities can foster healthier, more resilient communities equipped to navigate the complexities of academic life.

## Data Availability

The original contributions presented in the study are included in the article/supplementary material, further inquiries can be directed to the corresponding author.
